# Effects of Meteorological Factors on Daily Hospital Admissions for Asthma in Adults: A Time-Series Analysis

**DOI:** 10.1371/journal.pone.0102475

**Published:** 2014-07-14

**Authors:** Yue Zhang, Li Peng, Haidong Kan, Jianming Xu, Renjie Chen, Yuan Liu, Weibing Wang

**Affiliations:** 1 Department of Epidemiology, School of Public Health, and Key Laboratory of Public Health Safety (Ministry of Education), Fudan University, Shanghai, China; 2 Fudan Tyndall Centre, Fudan University, Shanghai, China; 3 Shanghai Center for Urban Environmental Meteorology, Shanghai, China; 4 Shanghai Key Laboratory of Meteorology and Health, Shanghai, China; University of Montana, United States of America

## Abstract

**Background:**

There is limited evidence for the impacts of meteorological changes on asthma hospital admissions in adults in Shanghai, China.

**Objectives:**

To quantitatively evaluate the short-term effects of daily mean temperature on asthma hospital admissions.

**Methods:**

Daily hospital admissions for asthma and daily mean temperatures between January 2005 and December 2012 were analyzed. After controlling for secular and seasonal trends, weather, air pollution and other confounding factors, a Poisson generalized additive model (GAM) combined with a distributed lag non-linear model were used to explore the associations between temperature and hospital admissions for asthma.

**Results:**

During the study periods, there were 15,678 hospital admissions for asthma by residents of Shanghai, an average 5.6 per day. Pearson correlation analysis found a significant negative correlation (r = −0.174, *P<0.001*) between asthma hospitalizations and daily mean temperature (DMT). The DMT effect on asthma increased below the median DMT, with lower temperatures associated with a higher risk of hospital admission for asthma. Generally, the cold effect appeared to be relatively acute, with duration lasting several weeks, while the hot effect was short-term. The relative risk of asthma hospital admissions associated with cold temperature (the 25^th^ percentile of temperature relative to the median temperature) was 1.20 (95% confidence interval [CI], 1.01∼1.41) at lag0-14. However, warmer temperatures were not associated with asthma hospital admissions.

**Conclusions:**

Cold temperatures may trigger asthmatic attacks. Effective strategies are needed to protect populations at risk from the effects of cold.

## Introduction

The World Health Organization (WHO) estimates that 300 million people worldwide have asthma and projects that an additional 100 million persons will have this disease by 2025 [1], [2]. Asthma accounts for about one percent of all disability-adjusted life years lost worldwide, reflecting the severity and prevalence of this disease [1]. The Global Initiative for Asthma (GINA) estimates that the prevalence of asthma in different countries ranges from 1% to 18% [3]. In China, the prevalence of asthma among adults has been reported to be around 2% [4], [5].

Asthma is a disease very much influenced by weather [6]. Climate change, caused in part by increased atmospheric concentrations of carbon dioxide (CO_2_) and other greenhouse gases, is likely to result in changes in temperature and humidity, and may relate to the increased burden of asthma [7]. Several studies have emphasized the relationships between low temperatures or cooling and asthmatic attacks. Cold temperature is a major environmental factor that exacerbates chronic inflammatory airway diseases (e.g., chronic obstructive pulmonary and asthma) [8]. In the short-term, cold temperatures are related to acute exacerbations of asthma symptoms, whereas warm temperatures are associated with increased asthma prevalence, due perhaps to higher levels of exposure to allergens [9], [10]. Some weather conditions including extremely hot and cold temperatures, changes in barometric pressure and humidity, and wind, can trigger asthmatic attacks [11], [12].

Weather conditions can affect the incidence of allergic respiratory diseases such as asthma by altering the dissemination of aeroallergens such as pollen and mold spores, with these effects emerging as a major indirect impact of climate change [13]. Climate warming can produce longer pollen seasons, whereas additional hot sunny summer days can generate ozone, with the two together increasing the symptoms in individuals at risk for asthma and breathing difficulties [14], [15], [16]. In addition, climate change can influence the concentrations of airborne pollutants, which, either alone or, in conjunction with aeroallergens, can exacerbate asthma and other respiratory illnesses [17].

Current knowledge of the effects of weather conditions on hospital admissions for respiratory diseases derives mainly from developed countries [18], [19]. Few studies have been conducted in developing countries, despite their being more sensitive to changes of weather conditions because of their poorer public health infrastructure and more vulnerable populations [20]. This study was therefore designed to evaluate the exposure-response relationship between hospital admissions for asthma and meteorological factors in an urban setting in China.

## Materials and Methods

### Data collection

Shanghai is located on the eastern tip of the Yangtze River Delta and along China's eastern coastline, at latitude 31°14′N and longitude 121°29′E. Shanghai is the most populous city in China, with a total population of over 23 million people in 2010. The city features a moderate subtropical climate, with four distinct seasons. The study population consisted of residents of Shanghai who participated in medical insurance for urban populations, including employees of urban businesses, organizations, institutions and social organizations.

Daily hospital admissions for asthma between January 1, 2005, and December 31, 2012, were collected from the Health Insurance System of Shanghai using the International Classification of Diseases Revision 10 (ICD 10) code J45. The Health Insurance System of Shanghai covers most of the residents in Shanghai (the coverage rate was 95% in 2008) and all hospitals are under contract with this System. Computerized records of hospital admissions are maintained at each contracted hospital and sent to the Health Insurance System through an internal computer network. Meteorological data on daily minimum, maximum and mean temperatures (°C), relative humidity (%), rainfall (mm) and wind speed (m/s) during the same period were obtained from the Shanghai Center for Urban Environmental Meteorology. The weather data were measured at a fix-site station located in Xuhui District of Shanghai. Air pollution data included particular matter less than or equal to 10 µm (PM_10_), sulfur dioxide (SO_2_) and nitrogen dioxide (NO_2_) during the same period were obtained from Shanghai Environmental Monitoring Center. The city-wide daily mean concentrations for each pollutant were averaged from the available data of six fixed-site monitoring stations (Hongkou, Jin'an, Luwan, Putou, Xuhui and Yangpu).

Patient records/information was de-identified prior to analysis; then daily aggregated counts for hospitalizations were calculated and used to conduct the final analysis. The authors did not have access to individual patient information prior to anonymization and data aggregation and there was no interaction with patients for this study.

### Data analysis

As the number of daily hospital admissions is a type of small probability event and typically follows a Poisson distribution [21], the semiparametric generalized additive model (GAM) approach with log link was used to explore the associations between daily mean temperature (DMT) and daily asthma admissions, accounting for any the over dispersion or autocorrelation.

In the first step, we utilized a time-series model to assess the relationship between DMT and hospital admissions for asthma, while controlling for relative humidity, rainfall and wind speed using a natural cubic spline. We also controlled the long-term trend and seasonal patterns using a natural cubic spline [22]. Day of the week (DOW) and public holidays (Holiday) were adjusted as dummy variables in the model, as these variables are potential confounders of the association between temperature and hospital admissions. The number of asthma hospital admissions was relatively small at the weekend and the holidays can also possibly affect hospitalization. Residuals of the basic models were used to check whether there were discernible patterns and autocorrelation by means of residual plots and partial autocorrelation function plots [23]. Additionally, for weather condition, the selection of degree of freedom was based on minimizing Akaike's Information Criterion (AIC). The basic model is as follows:
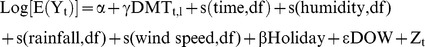
where Y_t_ refers to the number of the observation; E(Y_t_) denotes the estimated daily hospital admissions for asthma on day t; α is the intercept; γ is the vector of coefficients for DMT_t,l_, l is the number of lag days; S() denotes a regression spline function for nonlinear variables; time is the number of calendar days on day t; df is the degrees of freedom; Z_t_ is the independent variable for the linear effect on the dependent variable, here indicating the air pollutant concentrations of SO_2_, NO_2_ and PM_10_ on day t; DOW is the day of the week; and Holiday denotes public holidays.

Studies have shown that temperature can not only affect hospital admissions on that day but on several subsequent days (lag effect) [24], [25]. Previous studies have revealed that the relationship between temperature and respiratory diseases was non-linear [26]. Based on the core model, distributed lag non-linear models (DLNM) were developed to quantify the lag effects on health of daily mean temperature [27]. This method allows a cross-basis function to be defined as a combination of basic functions for two dimensions of DMT range and lag period. We constrained the DLNM models up to a lag of 30 days [28].

The DLNM allows showing the relationship between temperature and hospital admissions at each temperature point and lag. The median temperature was used as a reference value to calculate the relative risks. The 75^th^ (25.1°C), 25^th^ (9.4°C), 99^th^ (32.7°C), and 1^st^ (0.2°C) percentile of temperature relative to the median temperature were used to measure the risk of hot, cold, extremely hot and extremely cold, respectively.

All statistical analyses were performed using the R statistical environment (version 3.0.1) with the “DLNM” package used to fit the regression model.

## Results

Daily hospital admissions, environmental data and air pollutants from January 1, 2005, to December 31, 2012, are shown in Table 1. During this time period, totaling (2,922 days) 15,678 hospital admissions for asthma were recorded in Shanghai, a mean of 5.6 per day (range, 1–29). Admissions varied seasonally, with the peak number in winter, followed by autumn. During the study period, the DMT was 17.4°C (range, −3.4–35.7°C). The mean relative humidity (RH) was 69.5% (range, 23–95%), the mean rainfall was 31.2 mm (range, 0–1284 mm) and the mean wind speed (WS) was 3.0 m/s (range, 0.4–10.5 m/s). Mean concentration of SO_2_, NO_2_, PM_10_ were 41.8, 53.5 and 82.1 ug/m^3^, respectively.

**Table 1:** Summary statistics of daily asthma admissions, meteorological factors and air pollutants over 2922 days.

daily data	Mean	SD a	Min	p(1)	p(25)	Median	p(75)	p(99)	Max
**Asthma**	5.6	3.3	1.0	0.0	3.0	5.0	7.0	15.0	29.0
spring	5.2	3.1	1.0	0.0	3.0	5.0	7.0	15.0	24.0
summer	5.0	2.7	1.0	0.0	3.0	5.0	7.0	16.0	15.0
autumn	5.8	3.1	1.0	0.0	4.0	6.0	8.0	14.0	18.0
winter	6.6	3.9	1.0	0.0	4.0	6.0	9.0	18.0	29.0
**Meteorological factors**									
DMT(°C)	17.4	9.2	−3.4	0.2	9.4	18.7	25.1	32.7	35.7
DLT(°C)	14.3	9.3	−6.8	−3.6	6.3	15.1	22.5	29.1	31.8
DHT(°C)	21.1	9.4	−0.4	2.8	13.2	22.4	28.7	37.1	39.4
RH(°C)	69.5	12.3	23.0	38.0	62.0	70.0	79.0	92.0	95.0
JS(mm)	31.2	98.8	0.0	0.0	0.0	0.0	10.0	451.5	1284.0
WS(m/s)	3.0	1.1	0.4	1.2	2.3	2.9	3.6	6.1	10.5
**Air pollutants**									
SO_2_(ug/m^3^)	41.8	27.8	5.0	9.0	21.0	34.0	56.0	130.0	229.0
NO_2_(ug/m^3^)	53.5	21.7	6.0	14.0	38.0	51.0	66.0	118.8	155.0
PM_10_(ug/m^3^)	82.1	54.8	10.0	20.2	46.0	69.0	102.0	253.5	792.0

DMT: daily mean temperature; DLT: daily lowest temperature; DHT: daily highest temperature; RH: relative humidity; JS: rainfall; WS: wind speed.

a Standard deviation.

**Figure 1:**
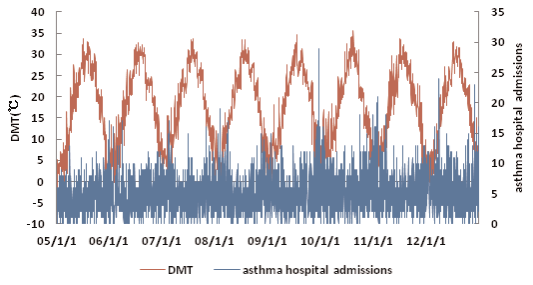
Daily mean temperature and daily hospital admissions for asthma over time in Shanghai, China during 2005 to 2012.

Hospital admissions for asthma and daily temperature over time are presented in Figure 1. Hospital admissions varied seasonally, peaking in winter (December to February). DMT showed significant periodicity, as well as random fluctuations. However when DMT was relatively low, the number of asthma hospital admissions appeared higher.

Pearson's correlations between daily hospital admissions for asthma, meteorological factors and air pollutants are shown in Table 2. Daily hospital admissions showed significant negative correlations with DMT (r = −0.174, *P<0.001*), rainfall (r = −0.044, *P<0.05*) and WS (r = −0.043, *P<0.05*). Daily hospital admissions for asthma showed significant positive correlations with SO_2_ (r = 0.039, *P<0.05*) and NO_2_ (r = 0.101, *P<0.001*). DMT showed significant positive correlations with relative humidity (r = 0.151, *P<0.001*), rainfall (r = 0.090, *P<0.001*) and WS (r = 0.099, *P<0.001*) and significant negative correlations with SO_2_ (r = −0.353, *P<0.001*), NO_2_ (r = −0.386, *P<0.001*) and PM_10_ (r = −0.175, *P<0.001*). Relative humidity showed a significant positive correlation with rainfall (r = 0.350, *P<0.001*), and reversely, significant negative correlations with SO_2_ (r = −0.326, *P<0.001*), NO_2_ (r = −0.186, *P<0.001*) and PM_10_ (r = −0.297, *P<0.001*).

**Table 2:** Pearson's correlations among asthma hospital admissions, meteorological factors and air pollutants.

	DMT	DHT	DLT	RH	JS	WS	SO_2_	NO_2_	PM_10_
asthma	−0.174**	−0.174 **	−0.170**	−0.016	−0.044*	−0.043*	0.039*	0.101**	0.014
DMT	1	0.984**	0.983**	0.151**	0.090**	0.099**	−0.353**	−0.386**	−0.175**
DHT		1	0.942**	0.072**	0.059**	0.073**	−0.290**	−0.312**	−0.109**
DLT			1	0.240**	0.121**	0.142**	−0.405**	−0.457**	−0.231**
RH				1	0.350**	−0.004	−0.326**	−0.186**	−0.297**
JS					1	0.122**	−0.162**	−0.131**	−0.145**
WS						1	−0.238**	−0.465**	−0.257**
SO_2_							1	0.724**	0.608**
NO_2_								1	0.661**
PM_10_									1

DMT: daily mean temperature; DLT: daily lowest temperature; DHT: daily highest temperature; RH: relative humidity;

JS: rainfall; WS: wind speed.

** *P<0.001*, * *P<0.05.*

**Figure 2:**
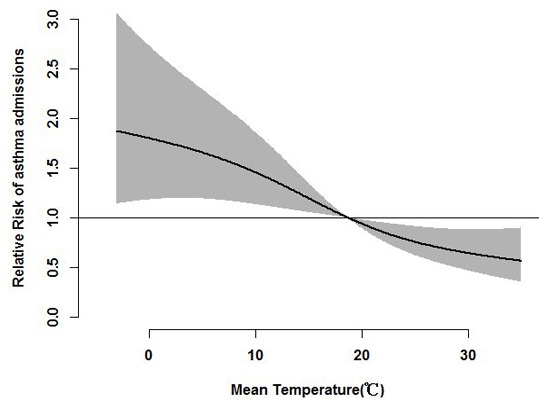
Overall effects of daily mean temperature on risk of asthma admissions over lag times of 0–30 days. The reference value was the median temperature (18.7°C).

Figure 2 shows the overall effects of DMT on hospital admissions for asthma for up to 30 days. Although no threshold level of DMT was associated with hospital admissions, admissions increased significantly below the median DMT of 18.7°C.

The cumulative effects of DMT on hospital admissions for asthma, including lag times, at the 1^st^, 25^th^, 75^th^, and 99^th^ percentiles of temperature relative to the median temperature are depicted in Table 3. When estimating the cumulative effects of cold temperature on asthma admissions, we found that cold temperature significantly increased the risks of hospital admissions at lag times of 14 to 30 days. For example, the relative risk of asthma hospital admissions associated with the 25^th^ percentile of temperature relative to the median temperature was 1.20 (95% confidence interval [CI], 1.01∼1.41) at lag 0–14. However, the association between high temperatures (the 75^th^ percentile of temperature relative to the median temperature) and asthma hospital admissions is limited (RR = 0.90, 95% confidence interval [CI], 0.80∼1.01)).

**Table 3:** Relative risk of asthma admissions associated with change in DMT between selected cutoff points.

Lag effects	1^st^ percentile relative to		25^th^ percentile relative to		75^th^ percentile relative to		99^th^ percentile relative to	
	median temperature		median temperature		median temperature		median temperature	
	RR	95%CI of RR	RR	95%CI of RR	RR	95%CI of RR	RR	95%CI of RR
0	1.06	(0.92,1.22)	1.03	(0.95,1.12)	1.00	(0.95,1.06)	1.06	(0.94,1.20)
0–1	1.08	(0.92,1.26)	1.03	(0.94,1.13)	1.02	(0.96,1.08)	1.10	(0.96,1.25)
0–2	1.11	(0.93,1.32)	1.04	(0.94,1.15)	1.01	(0.94,1.08)	1.06	(0.92,1.23)
0–3	1.07	(0.89,1.29)	1.01	(0.91,1.13)	1.03	(0.95,1.10)	1.09	(0.93,1.28)
0–7	1.23	(0.98,1.56)	1.09	(0.95,1.26)	0.98	(0.89,1.07)	0.99	(0.82,1.21)
0–14	1.35*	(1.02,1.79)	1.20*	(1.01,1.41)	0.90	(0.80,1.01)	0.83	(0.65,1.05)
0–21	1.53*	(1.09,2.15)	1.28*	(1.04,1.58)	0.83*	(0.71,0.97)	0.68*	(0.50,0.93)
0–30	1.79*	(1.18,2.72)	1.48*	(1.14,1.92)	0.75*	(0.62,0.91)	0.60*	(0.40,0.89)

DMT: daily mean temperature; RR: relative risk; CI: confidence interval.

*P-value<0.05;

1^st^ percentile: 0.2°C; 25^th^ percentile: 9.4°C; 75^th^ percentile: 25.1°C; 99^th^ percentile: 32.7°C.

Model included the following variables: the time trend, day of week, mean temperature, relative humidity, rainfall,

wind speed and air pollutants.

**Figure 3:**
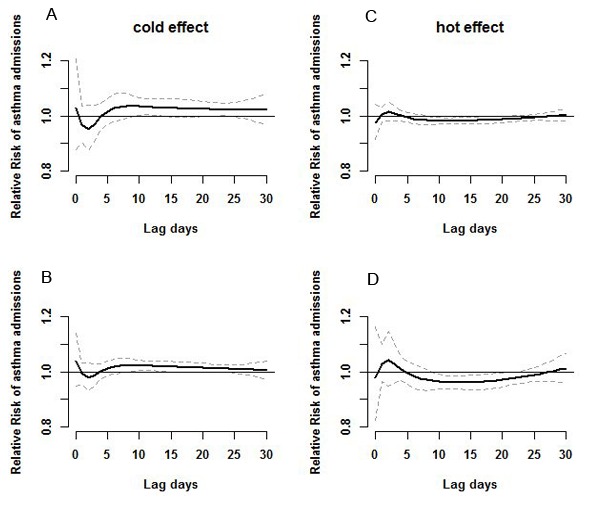
Estimated effects of cold and hot temperatures on hospital admissions for asthma over lag times of 0–30 days. Panel A shows the effect of 1^st^ percentile (0.2°C) relative to the median temperature(18.7°C); Panel B shows the effect of 25^th^ percentile (9.4°C) relative to the median temperature; Panel C shows the effect of 75^th^ percentile (25.1°C) relative to the median temperature; Panel D shows the effect of 99^th^ percentile (32.7°C) related to median temperature. The centre line in each graph shows the estimated spline curve of relative risk, and the upper and lower line show the 95% CIs.

Figure 3 shows the lag structure for relative risk of asthma hospital admissions associated with the quartiles of temperature relative to the median temperature according to the duration of lag time up to 30 days. We observed that the cold effects appeared to be relatively acute, with a duration lasting several weeks. The effect of cold temperature was higher than hot temperature. In contrast, the hot effects were relatively slower, with a lag time of 2–4 days.

## Discussion

To our knowledge, this study is the first to assess the effects of DMT on hospital admissions for asthma in China, over an 8-year period. We observed a statistically significant relationship between DMT and hospital admissions for asthma. Asthma hospital admissions varied seasonally, peaking in winter. The DMT effect increased significantly below the median temperature, consistent with previous findings [12]. In our analysis we found a 48% increase in asthma hospitalization with the 25^th^ percentile of temperature relative to the median temperature at lag 0–30. These results are consistent with a study conducted in eight cities in Koreas that found a significance in the association between asthma hospitalization and low temperature, with 43.6% increased at lag 0–32 [29]. In Shanghai, Guo et al. confirmed the association between asthma in children and cold temperature [30]. Cold temperature, which was related to exacerbation of respiratory diseases, has often been followed by an increase in bacterial and viral infections of the airways, infiltration of inflammatory factors, and mucus secretion [8], [31], [32]. Cold temperature has been associated with increased in the incidence of respiratory tract infections, and reduced temperatures often precedes the onset of infections [33], findings that may explain, at least in part, the effects of DMT on asthma.

We also observed that low temperatures had extended effects on hospital admissions for asthma, with lag periods of 14 through 30 lag days. The cold effect appeared to be relatively acute, lasting for several weeks. Although the relationship between DMT and asthma admissions had not previously been evaluated, several previous studies found that hot and cold temperatures has significant impacts on mortality rates in Shanghai. Moreover, there is a delay between changes in daily temperature and changes in the incidence and mortality from diseases, although the lag periods varied in different studies. Higher temperature were found to have short term effects on mortality and morbidity [34], [35], whereas the effects of low temperature were delayed and lasted for several days [18], [36]. An analysis in south China reported that hot temperatures had an acute but short-term effect, whereas the effect of cold temperatures lasted 10–12 days [37]. Similar findings were observed in Chiang Mai City, Thailand [38]. However, we failed to identify significance in the association between hot period and asthma hospital admissions. Some previous results reported the similar effects [29], [39]. While the cold effects from ambient temperature are clear, that of hotter temperature appears inconsistent. Because temperature affects many asthma risk factors that vary in prevalence and seasonality by region, patterns of association between temperature and asthma are also expected to differ geographically [40]. The magnitude and direction of the effects of temperature on asthma hospital admissions may be related to differences in the levels of exposure, susceptibility of subpopulations, public health interventions, health and social care services, and physical acclimatization [41], [42].

This finding suggests that a longer time frame is required to capture the effects of cold, and that it may be inappropriate to specify an identical time frame for exposure to cold and hot temperatures. We investigated the effects of temperature on hospital admissions for asthma using a sophisticated statistical approach. As the dose-response curve relating these two factors is not exactly linear below and above a threshold, it is inappropriate to use linear threshold models to directly estimate these effects. DLNMs, which unify many previous methods in one unique framework, are flexible enough to describe non-linear dependencies and delayed effects of exposure at the same time [27]. DLNMs can be easily translated into other study designs and regression models [43]. In the present study, we fitted DLNMs to fully understand the dose-response function and lag effects of temperature.

This study had several limitations. Firstly, we use weather conditions at one meteorological station as measurements of DMT rather than measures of personal exposure. The use of ambient rather than personal exposure measures may result in exposure misclassification. Moreover, the temperature difference between indoors and outdoors due to air conditioning or heating may affect the association between temperature and asthma. Secondly, the data on asthma hospital admissions were collected from the Shanghai Health Insurance System, thus possibly introducing a selection bias. Thirdly, daily mean concentrations of air pollutants may vary more than daily mean temperature, which leads to limited ability to account for local differences in air pollutants with respect to asthma hospital admissions. This might reduce the power of the study, but it is not likely to attenuate the risk estimates. Finally, although the occurrence of asthma has been reported to be closely related to age and gender [44], [45], [46], this study did not use the models for subgroup analysis.

## Conclusions

This study demonstrated that hospital admissions for asthma were significantly associated with DMT below the median temperature. The effects of low temperature on hospital admissions showed a lag effect, lasting from 14 days to 30 days. The cold effect appeared to be relatively acute and lasted for several weeks. The findings suggest that cold temperature may trigger asthma attacks and that effective strategies are needed to protect populations at risk from the effects of cold.
